# Examining racial disparity in psychotic disorders related ambulatory care visits: an observational study using national ambulatory medical care survey 2010–2015

**DOI:** 10.1186/s12888-023-05095-y

**Published:** 2023-08-17

**Authors:** Shahrzad Bazargan-Hejazi, Anaheed Shirazi, David Hampton, Deyu Pan, Daniel Askharinam, Magda Shaheen, Gul Ebrahim, Denese Shervington

**Affiliations:** 1 Department of Psychiatry, College of Medicine, Charles R. Drew University of Medicine and Science and UCLA David Geffen School of Medicine, Los Angeles, CA USA; 2 Department of Psychiatry, School of Medicine, University of California San Diego, San Diego, CA USA; 3 Department of Family Medicine, College of Medicine, Charles R. Drew University of Medicine and Science, Los Angeles, CA USA; 4 Charles R Drew University of Medicine and Science, Los Angeles, CA USA; 5 Kedren Community Health Care Center, Los Angeles, CA USA; 6 College of Medicine, Charles R. Drew University of Medicine and Science, UCLA David Geffen School of Medicine, Los Angeles, CA USA; 7 Kedren Community Care Clinic and Charles R Drew University of Medicine and Science, Los Angeles, CA USA; 8 Department Psychiatry, College of Medicine, Charles R. Drew University of Medicine and Science, Los Angeles, CA USA

**Keywords:** Racial disparity, Psychotic Disorders, Ambulatory care visits

## Abstract

**Background:**

One of the most consistent research findings related to race and mental health diseases is the disproportionately high rate of psychotic disorder diagnoses among people of color, specifically people of African descent. It is important to examine if a similar pattern exists among specific psychotic disorders. We aimed to examine the racial/ethnic differences in ambulatory care visits diagnosed with schizophrenia-spectrum disorders (SSDs).

**Methods:**

We analyzed data from the National Ambulatory Medical Care Survey (NAMCS) 2010–2015. The study sample included physician office-based visits by individuals diagnosed with SSDs, including schizophrenia, schizoaffective, and unspecified psychotic disorder (n = 1155). We used descriptive and bivariate analysis by race/ethnicity and three multinomial logistic regression models to test the association between the SSDs and race/ethnicity, adjusting for age, gender, insurance, disposition, medication Rx, and co-morbidity, considering the design and weight.

**Result:**

Of the 1155 visits for SSDs, 44.8% had schizophrenia, 37.4% had schizoaffective disorder diagnosis, and 19.0% had unspecified psychosis disorder. We found significant racial disparities in the diagnosis of SSDs. Black patients were overrepresented in all three categories: schizophrenia (24%), schizoaffective disorder (17%), and unspecified psychosis disorder (26%). Also, a notable percentage of Black patients (20%) were referred to another physician in cases of schizophrenia compared to other ethnoracial groups (p < 0.0001). Moreover, we found a significant disparity in insurance coverage for schizoaffective disorder, with a higher percentage of Black patients (48%) having Medicaid insurance compared to patients from other ethnoracial groups (p < 0.0001). Black patients had nearly twice the odds of receiving a diagnosis of schizophrenia compared to White patients [AOR = 1.94; 95% CI: 1.28–2.95; P = 0.001]. However, they had significantly lower odds of being diagnosed with schizoaffective disorder [AOR = 0.42, 95% CI: 0.26–0.68; P = 0.003]. Race/ethnicity was not associated with receiving an unspecified psychosis disorder.

**Conclusions:**

Our results show that SSDs, more specifically schizophrenia, continue to burden the mental health of Black individuals. Validation of our findings requires rigorous research at the population level that reveals the epidemiological difference of SSDs diagnoses in different race/ethnicity groups. Also, advancing our understanding of the nature of disparity in SSDs diagnoses among the Black population requires disentangling etiologic and systemic factors in play. This could include psychological stress, the pathway to care, services use, provider diagnostic practice, and experiencing discrimination and institutional and structural racism.

## Introduction

Schizophrenia is a chronic mental health disorder that affects between 0.3 and 1.6% of the population in the United States (US) [1, 2]. Due to its chronic nature and onset in young adulthood, schizophrenia is among the fifteen leading causes of disability and is associated with a substantial economic burden [3]. In the US, the costs associated with schizophrenia have steadily increased since 1975, from $11.6 billion to 155.7 billion dollars in 2013 [4, 5].

One of the most consistent research findings from the US and European studies related to minority and mental health diseases is the disproportionately high rate of schizophrenia-spectrum disorders (SSDs) and other psychotic disorders among ethnic minorities, specifically Black patients [6–13]. These studies show apparent ethnic disparities in the rate of SSDs and other psychotic disorders in the population. They reveal the complexity and varied epidemiological landscape for the prevalence and incidence of psychotic disorders and the role of socio-psychological and socio-environmental factors in the etiology of schizophrenia [8, 12, 13]. For example, it has been highlighted that the lack of social capital as an environmental risk factor increases the risk of psychotic disorders in the minoritized ethnoracial groups [14–17]. Others argue that the clinical presentation and the general psychopathology of schizophrenia and psychosis, especially among Black patients, may implicate ethnicity’s role in the initial symptoms’ treatment path [6]. Several empirical evidence from US-based studies have reported that Black patients demonstrate more severe psychotic symptoms [18], and first-rank symptoms, suggesting racial disparity in diagnosis may be secondary to more severe first-rank symptoms in this population [19]. These investigators suggest that evaluating psychotic symptoms in the context of other symptoms, such as affective symptoms, could prevent misdiagnoses of schizophrenia [19–21]. Additional empirical studies have sought other explanations for observed ethnoracial differences in psychotic disorders. These studies suggest that ethnoracial differences could be influenced by differences in the pathway to care, services use, and provider diagnostic bias [21–23] psychosocial stressors, including childhood trauma, stressful life events, experiencing discrimination, and institutional and structural racism [10, 24–27].

Since schizophrenia is one of several specific psychotic disorders demonstrating race-specific disparities, it is important to consider whether a similar pattern exists among other psychotic disorders [25]. Disorders with psychotic features range from schizophrenia to more complex diseases such as schizoaffective disorder, to shorter duration disorders such as schizophreniform disorder, to disorders with narrow symptomatology such as delusional disorder, and mood disorders with psychotic features [28]. In the current study, we aimed to examine the racial/ethnic differences in ambulatory care visits diagnosed with schizophrenia-spectrum disorders, including schizophrenia, schizoaffective disorder, and unspecified psychotic disorder.

## Methods

### Study design and database

This observational study analyzed data from the National Ambulatory Medical Care Survey (NAMCS) 2010–2015. The NAMCA is a national probability sample survey of visits to office-based physicians who were not federally employed and were primarily providing patient care. Further details are available from the National Center for Health Statistics [29]. The data can also be weighted to produce national estimates that describe the utilization of ambulatory medical care services in the US. Of the 4,910 in-scope (eligible) physicians, 1,410 participated by submitting data for patient visits using a standardized survey (patient record form) that either the clinician or the other staff completed during a 1-week reporting period after the patient visit. Data for this study were obtained from 270,346 visits from 2010 to 2015.

### Eligibility criteria and study sample

SSDs-related office-based physician visits were identified from the sample of all physician visits based on a primary diagnosis listed by the treating physician with ICD-9 codes 295 and 298.9 (Three diagnoses between 2010 and 2013, five diagnoses in 2014 and 2015). The study sample included visits by individuals diagnosed with SSDs, including schizophrenia, schizoaffective, and unspecified psychosis disorder, and had no missing data in the SSDs and race/ethnicity variables (n = 1155).

### Study measures

The outcome variable, SSDs, were categorized as [1] schizophrenia (ICD-9 295.0, 295.1, 295.2, 295.3, 295.4, 295.5, 295.6, 295.9); [2] schizoaffective disorder (ICD-9 295.7); and [3] unspecified psychosis disorder (ICD-9 298.9). Race/ethnicity as the primary predictor was measured as White, Black, Hispanic, and Asian/Others including Asian, Native Hawaiian/Other Pacific Islander, American Indian or Alaska Native, and Multiple ethnic groups. Other independent variables included in the study were age groups (18–44, 45–64, and 65 years and above), gender (female and male), insurance status (private, Medicare, Medicaid, and others including worker’s compensation, self-pay, no charge/charity), visit disposition (categorized as refer to other physicians, return at a specified time, and other visit disposition including ER and hospital), and medication Rx (categorized as receiving psychotropic agents -monotherapy or combination, antidepressants, and antipsychotics). We also included physical co-morbidities in the list of independent variables because of the existing evidence suggesting the prevalence of physical comorbidities in schizophrenia patients is high compared to their general population [30], which could have implications for its treatment (For example, treatment with clozapine) [31] and healthcare utilization from the side of patients, caregivers, and providers [32–34]. In our study, co-morbidity covered lung diseases (asthma, chronic obstructive pulmonary disease [COPD], cardiovascular disease [CVD], hyperlipidemia), depression, diabetes, hypertension, and other co-morbidities [including arthritis, cancer, chronic renal failure, congestive heart failure, obesity, and osteoporosis]. Depression diagnosis was identified if the provider marked “x” in the question “Regardless of the diagnoses written, does the patient now have depression?”[35].

### Statistical analysis

SSDs-related office visits to physicians were the units of analysis. Descriptive statistics were used to depict the characteristics of the visits and were presented as unweighted numbers and weighted percentages for categorical data. Additionally, we divided a weighted number of visits for SSDs by the number of people (age group or gender group, or particular race/ethnicity group) in the US in 2010. We presented as a percentage rate per year. We used the listwise deletion option in the analysis for the missing data for the other independent variables. We used Chi-square tests to examine the relationship between the outcome variables (schizophrenia, schizoaffective disorder, unspecified psychosis disorder) and study variables with race/ethnicity. We conducted post hoc analysis, adjusted for the experiment-wise error, and also adjusted the p-value using the formula 1 – (1 – α)^3^ = 0.05. With six separate tests, to achieve a combined type I error rate (an experiment-wise error rate) of 0.05, we set each alpha to a value using α = 1 – (1 – 0.05)^1/3^ = 0.01. The proportion of patients with reported depression were compared across the racial/ethnic groups using the Chi-square test.

We used three multinomial logistic regression models to test the association between the SSDs and race/ethnicity, adjusting for the other independent variables in the models. The other independent variables included in the model were selected in advance based on the literature review and discussion with experts in the field. In the multinomial logistic regression models, visits for schizophrenia (coded as 1) were compared with visits by patients who had the other two diagnoses (combined schizoaffective diagnosis and unspecified psychosis disorder [coded as 0]), same for the schizoaffective and unspecified psychosis diagnoses.

Data were presented as adjusted odds ratio (AOR) and 95% confidence interval (CI). A p-value < 0.01 was considered statistically significant. SAS version 9.3 was used to analyze the data. We used the sample weights provided by the NAMCS to correct differential selection probabilities and to adjust for non-coverage and non-response.

## Results

### Descriptive

Table 1 presents descriptive statistics (weighted percentages) for 1155 visits for SSDs: schizophrenia, schizoaffective, and unspecified psychosis disorder. As indicated, of the visits, 61.8% were White patients, 21.2% were Black patients, 11.4% were Hispanic patients, and Asian/other groups made up 5.7%. Among the visits, 56.0% were males, 48.5% were in the 45–64 age group, 44.8% received care for schizophrenia diagnosis, 37.4% for schizoaffective disorder care, and 19.0% for unspecified psychosis disorder. Moreover, 25.6% (n = 300) of the patients had co-morbid depression, 82.0% used some psychotropic agents (monotherapy or combination), 39.4% (n = 456) were prescribed antidepressants, and 75.8% (n = 886) were prescribed antipsychotic medications (Table 1). Additionally, based on our percent rate calculation, the rate of SSDs was higher in the 45–64 years old group [1.71%], male [1.34%], and Black patients [2.08%] (Table 2).

**Table 1:** Ambulatory care visits’ characteristics for patients diagnosed with Schizophrenia-Spectrum Disorders (SSDs): Data from the NAMCS 2010–2015 (N = 1155)

Variable	Number of visits	Weighted %
**Age**		
• 18–44 • 45–64 • 65+	477 520 158	**37.4** **48.5** **14.1**
**Gender**		
• Female • Male	493 662	**43.7** **56.3**
**Race/ethnicity**		
• White • Black • Hispanic • Asian/Other identies	700 253 129 73	**61.8** **21.2** **11.4** **5.6**
**Insurance**		
• Private • Medicare • Medicaid • Other	162 465 334 194	**15.0** **39.1** **27.0** **18.9**
**Psychotic Disorders (% Yes)**		
• Schizophrenia Dx • Schizoaffective Dx • Unspecified psychosis Dx	526 415 230	**44.8** **37.4** **19.0**
**Co-morbidity (% Yes)**		
• Lung Dx (Asthma, COPD) • CVD (CVD, Hyperlipidemia) • Depression • Diabetes • Hypertension • Other Co-morbidities*	79 151 300 124 236 180	**7.9** **13.6** **25.6** **11.3** **23.5** **15.2**
**Disposition (% Yes)**		
• Refer to another physician • Return at specified time/Follow up • Another visit disposition (include ER and Hosp)	59 1024 117	**6.3** **90.1** **8.6**
**Medication Use (% Yes)**		
• Psychotropic Rx (monotherapy or combination) • Antidepressants • Antipsychotics	944 456 886	**82.1** **39.4** **75.8**

*Other comorbidities include: Arthritis, Cancer, Chronic renal failure, Congestive heart failure, Obesity, Osteoporosis

**Table 2:** Rate of ambulatory care visits for patients diagnosed with Schizophrenia-Spectrum Disorders (SSDs): Data from the NAMCS 2010–2015 (N = 1155)

Variable	Number of Visits	Weight number	Rate (%)
**Age**			
• 18–44 • 45–64 • 65+	477 520 158	6,106,822 8,836,434 1,577,997	0.89 1.71 0.65
**Gender**			
• Female • Male	493 662	7,098,818 9,422,435	0.95 1.34
**Race/ethnicity** • White	700 253	10,220,332 3,584,310	1.07 2.08
• Black • Hispanic • Asian/other	129 73	1,811,621 904,990	0.84 0.84

### Bivariate associations between race/ethnicity and schizophrenia spectrum disorders (SSDs)

#### Schizophrenia

As illustrated in Table 3, among patients diagnosed with schizophrenia, 57.9% were White, 23.9% were Black, 11.4% were Hispanic, and 6.6% were Asian or belonged to another ethno-racial group.

Table 3 also shows an association between race/ethnicity and several demographic and clinical variables including insurance, disposition, medication Rx, and co-morbidity (lung, asthma, COPD, and diabetes) among patients with schizophrenia (p = < 0.01). More specifically, among Black patients with schizophernia, 43.3% had Medicaid insurance compared to 29.7% among White patients, 29.6% among Hispanic patients, and 13.3% among Asian/Others group (p = 0.004). Slightly over 20% of Black patients were referred to another physician compared to 4.5% of the White patients, 5.5% of the Hispanic patients, and 0.02% among Asian/Others group (p < 0.0001). Over 65% of the Black patients (65.3%) received psychotropic agent such as monotherapy or combinations compared to 78.6% of the White patients, 79.8% of the Hispanic patients, and 90.5% of the Asian/Others group (p = 0.0111). Additionally, 13.2% of the Black patients presented with lung, asthma, COPD diseases compared to 9.3% among White patients, 1.8% among Hispanic patients, and 1.7% among Asian/Others group (p = 0.0084). Moreover, 41.3% of Black patients presented with diabetes compared to 20% among White patients, 21.1% of Hispanic patients, and 10.7% among the Asian/Others group (p = 0.0006).

### Schizoaffective diagnosis

Of the visits for schizoaffective diagnosis, 65.7% were White patients, 17.1% were Black patients, 10.6% were Hispanic patients, and 6.6% belonged to Asians/Others group. Among these visits, we found a significant association between insurance and race/ethnicity (p = < 0.01). More specifically, among Black patients, 48.3% had Medicaid insurance compared to 17.1% among White patients, 32.3% among Hispanic patients, and 19.3% among Asian/Others group (p = 0.003).

### Unspecified psychosis disorder

Visits for unspecified psychosis disorder were made by 57.3% of Whites patients, 26.0% of Black patients, 11.3% of Hispanic patients, and 5.2% of Asians/Others. Among these visits, we found a significant association between gender and race/ethnicity (p = < 0.01). More specifically, among Black patients, 58.1% were males compared to 43.8% among White, 58.0% among Hispanics, and 94.9% among Asian/Others (p = 0.009).

### Independent relationship between race/ethnicity and visits for schizophrenia, schizoaffective, and unspecified psychosis disorder

Table 4 illustrates three multinomial logistic regression models examining the independent relationship of race/ethnicity and ambulatory visits for schizophrenia, schizoaffective, and unspecified psychosis disorder while controlling for age, gender, insurance, disposition, medication Rx, and co-morbidity. Our results revealed that compared to diagnosis received by White patients, Black patients had nearly twice the odds of receiving a schizophrenia diagnosis [AOR = 1.94; 95% CI: 1.28–2.95; P = 0.001], however, they had 0.42 times odds of visiting for schizoaffective disorder diagnosis [AOR = 0.42, 95% CI: 0.26–0.68; P = 0.003]. Race/ethnicity was not associated with receiving unspecified psychosis disorder.

**Table 3:** Racial/ethnic variation in the ambulatory care visits for patients diagnosed with Schizophrenia-Spectrum Disorders (SSDs), including Schizophrenia, Schizoaffective and Unspecified Psychosis Disorder

	Schizophrenia					
	Total Visits N (%) 526 (44.8)	White Visits n (%) 305 (57.9)	Black Visits n (%) 126 (23.9)	Hispanic Visits n (%) 60 (11.4)	Asian/Others Visits n (%) 35 (6.6)	
	*n (weighted %)*	*n (weighted %)*	*n (weighted %)*	*n (weighted %)*	*n (weighted %)*	p-value
**Age**						0.1123
• 18–44 • 45–64 • 65+	199 (33.3%) 262 (53.6%) 65 (13.1%)	106 (34.2%) 163 (53.3%) 36 (12.6%)	49 (25.1%) 62 (63.3%) 15 (11.7%)	26 (46.1%) 25 (41.2%) 9 (12.7%)	18 (44.4%) 12 (28.9%) 5 (26.7%)	
**Gender**						0.9553
• Female • Male	183 (34.9%) 343 (65.2%)	111 (35.5%) 194 (64.5%)	38 (32.3%) 88 (67.7%)	24 (36.2%) 36 (63.8%)	10 (38.7%) 25 (61.3%)	
**Insurance**						0.0041
• Private • Medicare • Medicaid • Other	57 (10.2%) 204 (37.8%) 177 (32.7%) 88 (19.3%)	34 (10.9%) 132 (44.1%) 96 (29.7%) 43 (15.2%)	11 (9.5%) 39 (23.9%) 54 (43.3%) 22 (23.4%)	6 (11.1%) 20 (36.9%) 21 (29.6%) 13 (22.4%)	6 (4.8%) 13 (51.0%) 6 (13.3%) 10 (30.9%)	
**Disposition (% Yes)**						
• Refer to another physician • Return at specified time • Other visit disposition	28 (9.3%) 468 (89.7%) 50 (8.4%)	18 (4.5%) 272 (90.1%) 29 (10.6%)	6 (21.2%) 105 (85.0%) 17 (7.2%)	3 (5.5%) 57 (97.5%) 3 (2.5%)	1 (0.02%) 34 (95.2%) 1 (4.8%)	< 0.0001 0.0541 0.1572
**Medication use (% Yes)** • Psychotropic Rx (monotherapy or combination) • Antidepressants • Antipsychotics	413 (75.5%) 166 (29.9%) 398 (72.6%)	240 (78.6%) 98 (30.9%) 230 (75.7%)	97 (65.3%) 40 (28.4%) 93 (61.0%)	46 (79.8%) 22 (37.3%) 46 (61.0%)	30 (90.9%) 6 (14.3%) 29 (90.5%)	0.0111 0.2441 0.0026
**Co-morbidity (% Yes)**						
• Lung, Asthma, COPD • CVD, Hyperlipidemia • Depression • Diabetes • Hypertension • Other Co-morbidities	37(9.2%) 86 (18.0%) 122 (20.1%) 58 (9.7%) 115 (25.8%) 80 (16.4%)	25 (9.3%) 49 (14.9%) 78 (19.0%) 26 (6.3%) 57 (20.0%) 44 (13.2%)	9 (13.2%) 24 (25.5%) 27 (25.4%) 18 (13.3%) 39 (41.3%) 23 (26.3%)	1 (1.8%) 10 (18.5%) 13 (16.1%) 10 (17.5%) 14 (21.1%) 7 (9.0%)	2 (1.7%) 3 (7.6%) 4 (11.3%) 4 (8.3%) 5 (10.7%) 6 (10.6%)	0.0084 0.0839 0.3153 0.0437 0.0006 0.0259
	**Schizoaffective Dx**					
	**Total Visits** **N (%)** **415 (37.4)**	**White Visits** **n (%)** **273 (65.7)**	**Black Visits** **n (%)** **71 (17.1)**	**Hispanic Visits** **n (%)** **44 (10.6)**	**Asian/Others Visits** **n (%)** **27 (6.6)**	**p-value**
	***n (weighted %)***	***n (weighted %)***	***n (weighted %)***	***n (weighted %)***	***n (weighted %)***	
**Age**						0.0162
• 18–44 • 45–64 • 65+	190 (41.0%) 190 (50.3%) 35 (8.6%)	126 (39.5%) 119 (50.9%) 28 (9.7%)	30 (39.5%) 40 (59.6%) 1 (0.9%)	18 (39.6%) 22 (50.6%) 4 (9.8%)	16 (68.4%) 9 (21.4%) 2 (10.2%)	
**Gender**						0.2372
• Female • Mal	215 (53.5%) 200 (46.5%)	145 (56.5%) 128 (43.5%)	37 (44.2%) 34 (55.8%)	18 (42.7%) 26 (57.3%)	15 (56.8%) 12 (43.2%)	
**Insurance**						0.0030
• Private • Medicare • Medicaid • Other	58 (15.5%) 179 (43.2%) 115 (22.7%) 63 (18.7%)	50 (18.9%) 118 (45.2%) 65 (17.1%) 40 (18.8%)	1 (0.9%) 31 (34.0%) 28 (48.3%) 11 (16.8%)	4 (8.3%) 17 (42.4%) 14 (32.2%) 9 (17.1%)	3 (17.5%) 13 (39.0%) 8 (19.3%) 3 (24.1%)	
**Disposition (% Yes)**						
• Refer to another physician • Return at specified time • Other visit disposition	20 (3.2%) 373 (93.8%) 38 (6.4%)	14 (3.1%) 243 (94.3%) 26 (5.9%)	3 (3.3%) 65 (93.0%) 6 (6.3%)	1 (0.6%) 41 (96.5%) 4 (8.7%)	2 (9.2%) 24 (83.9%) 2 (9.1%)	0.1092 0.2026 0.1572
**Medication (% Yes)**						
• Psychotropic Rx (monotherapy combination) • Antidepressants • Antipsychotics	373 (92.1%) 206 (50.0%) 351 (85.0%)	239 (90.3%) 134 (47.3%) 225 (82.5%)	66 (97.6%) 36 (54.0%) 63 (92.2%)	42 (97.9%) 21 (56.4%) 38 (90.0%)	26 (91.6%) 15 (63.6%) 25 (90.9%)	0.1365 0.4005 0.3635
**Co-morbidity (% Yes)**						
• Lung, Asthma, COPD) • CVD, Hyperlipidemia • Depression • Diabetes • Hypertension • Other Co-morbidities	33 (7.6%) 38 (8.5%) 126 (32.1%) 42 (11.4%) 75 (17.8%) 72 (15.1%)	21 (6.6%) 24 (7.1%) 82 (30.0%) 18 (8.6%) 43 (15.3%) 48 (14.4%)	5 (8.2%) 5 (8.8%) 22 (39.5%) 12 (16.9%) 17 (28.0%) 11 (15.5%)	4 (11.5%) 6 (15.1%) 15 (32.8%) 9 (22.4%) 13 (26.6%) 5 (9.4%)	3 (10.7%) 3 (11.4%) 7 (41.7%) 3 (14.2%) 2 (9.0%) 8 (34.8%)	0.6616 0.4198 0.6296 0.0626 0.0346 0.0603
	**Unspecified Psychosis Disorder**					
	**Total Visits** **N (%)** **230 (19.0)**	**White Visits** **n (%)** **132 (57.3)**	**Black Visits** **n (%)** **60 (26.0)**	**Hispanic Visits** **n (%)** **26 (11.3)**	**Asian/Others Visits** **n (%)** **129 (5.2)**	**p-value**
	***n (weighted %)***	***n (weighted %)***	***n (weighted %)***	***n (weighted %)***	***n (weighted %)***	
**Age**						0.1518
• 18–44 • 45–64 • 65+	94 (38.9%) 75 (34.2%) 61 (26.9%)	49 (38.0%) 41 (31.6%) 42 (30.4%)	29 (48.4%) 21 (35.2%) 10 (16.4%)	11 (37.5%) 8 (29.2%) 7 (33.3%)	5 (20.9%) 5 (65.9%) 2 (13.1%)	
**Gender**						0.0009
• Female • Male	106 (48.2%) 124 (51.8%)	72 (56.2%) 60 (43.8%)	21 (41.9%) 39 (58.1%)	10 (42.0%) 16 (58.0%)	3 (5.1%) 9 (94.9%)	
**Insurance**						0.0254
• Private • Medicare • Medicaid • Other	49 (26.4%) 86 (33.1%) 48 (21.5%) 47 (19.0%)	35 (27.5%) 44 (33.5%) 27 (23.8%) 26 (15.3%)	6 (13.9%) 30 (38.3%) 12 (17.6%) 12 (30.2%)	4 (20.3%) 10 (37.6%) 6 (21.1%) 6 (21.0%)	4 (66.5%) 2 (4.5%) 3 (13.6%) 3 (15.3%)	
**Disposition (% Yes)**						
• Refer to another physician • Return at specified time • Other visit disposition	12 (5.4%) 197 (83.4%) 30 (13.1%)	8 (7.2%) 110 (80.5%) 19 (12.2%)	3 (2.5%) 55 (91.4%) 4 (6.1%)	1 (4.1%) 23 (83.8%) 3 (26.2%)	0 (0.0%) 9 (85.5%) 4 (16.8%)	0.1187 0.2873 0.1278
**Medication Use (% Yes)**						
• Psychotropic Rx (monotherapy or combination) • Antidepressants • Antipsychotics	172 (78.2%) 89 (40.3%) 151 (65.9%)	94 (75.1%) 54 (39.3%) 79 (62.0%)	47 (78.4%) 19 (39.5%) 45 (73.7%)	22 (89.8%) 12 (49.1%) 18 (63.4%)	9 (84.2%) 4 (34.0%) 9 (84.2%)	0.3153 0.8661 0.3172
**Co-morbidity (% Yes)**						
• Lung, Asthma, COPD) • CVD, Hyperlipidemia • Depression • Diabetes • Hypertension • Other Co-morbidities	10 (5.4%) 30 (13.2%) 58 (28.1%) 28 (16.2%) 49 (28.9%) 30 (12.1%)	7 (6.6%) 19 (14.1%) 35 (27.7%) 17 (20.4%) 28 (28.8%) 15 (12.2%)	2 (4.7%) 7 (9.0%) 12 (26.5%) 6 (14.8%) 11 (15.9%) 11 (17.0%)	1 (3.4%) 3 (21.0%) 9 (36.1%) 3 (4.8%) 7 (32.3%) 2 (6.9%)	0 (0.0%) 1 (2.3%) 2 (20.1%) 2 (4.5%) 3 (62.8%) 2 (7.4%)	0.5026 0.4295 0.8596 0.0291 0.1283 0.6943

**Table 4:** Adjusted odds ratio (AOD) and 95% confidence interval of the factors associated with ambulatory care visits for Schizophrenia-Spectrum Disorders (SSDs), including Schizophrenia, Schizoaffective, and Unspecified psychosis disorder, NAMCS 2010–2015

Outcome	Model 1 outcome Schizophrenia (N = 526)		Model 2 outcome Schizoaffective (N = 415)		Model 3 outcome Unspecified psychosis disorder (N = 230)	
Variable	AOD (95% CI)	p-value	AOD (95% CI)	p-value	AOD (95% CI)	p-value
**Age**						
• 18–44 (Ref)						
• 45–64	1.52 (1.16–1.97)	0.0020	0.90 (0.68–1.20)	0.4875	0.60 (0.40–0.88)	0.0093
• 65+	1.34 (0.67–2.67)	0.4077	0.29 (0.15–0.56)	0.0002	2.44 (1.34–4.44)	0.0035
**Gender**						
• Female (Ref)						
• Male	1.81 (1.31–2.51)	0.0003	0.52 (0.39–0.69)	< 0.0001	0.87 (0.59–1.26)	0.4537
**Race/ethnicity**						
• White (Ref)						
• Black	1.94 (1.28–2.95)	0.0019	0.42 (0.26–0.68)	0.0003	1.18 (0.65–2.16)	0.5889
• Hispanic	1.20 (0.77–1.87)	0.4317	0.74 (0.46–1.18)	0.2022	1.27 (0.76–2.13)	0.3559
• Asian/other	1.34 (0.85–2.13)	0.2097	0.68 (0.32–1.46)	0.3247	1.11 (0.49–2.53)	0.8004
**Insurance**						
• Private (Ref)						
• Medicare	1.93 (1.19–3.14)	0.0080	1.30 (0.75–2.25)	0.3449	0.24 (0.13–0.43)	< 0.0001
• Medicaid	2.62 (1.53–4.48)	0.0005	0.82 (0.45–1.47)	0.4974	0.31 (0.17–0.55)	<0.0001
• Other	1.61 (0.90–2.86)	0.1064	1.18 (0.61–2.30)	0.6219	0.39 (0.20–0.75)	0.0047
**Disposition**						
• Refer to another physician						
-No (Ref)						
-Yes	3.28 (1.81–5.93)	<0.0001	0.53 (0.29–0.97)	0.0379	0.35 (0.14–0.89)	0.0268
• Return at specified time						
-No (Ref)						
-Yes	1.47 (0.77–2.79)	0.2464	1.64 (0.82–3.25)	0.1601	0.24 (0.09–0.61)	0.0030
• Other visit disposition						
-No (Ref)						
-Yes	1.19 (0.56–2.50)	0.6535	1.09 (0.57–2.10)	0.7934	0.49 (0.18–1.31)	0.1524
**Medication use **						
• Psychotropic Rx (monotherapy or combination)						
-No (Ref)						
-Yes	0.22 (0.10–0.49)	0.0002	2.62 (0.99–6.94)	0.0526	1.88 (0.81–4.36)	0.1412
• Antidepressants						
-No (Ref)						
-Yes	0.59 (0.41–0.85)	0.0046	1.43 (0.99–2.06)	0.0586	1.20 (0.76–1.90)	0.4329
• Antipsychotics						
-No (Ref)						
-Yes	2.71 (1.46–5.06)	0.0017	1.13 (0.57–2.27)	0.7236	0.33 (0.18–0.63)	0.0007
**Co-morbidity**						
• Lung, Asthma, COPD						
-No (Ref)						
-Yes	1.66 (0.98–2.80)	0.0576	0.90 (0.57–1.43)	0.6565	0.53 (0.29–0.99)	0.0463
• CVD, Hyperlipidemia						
-No (Ref)						
-Yes	2.68 (1.62–4.45)	0.0001	0.44 (0.27–0.72)	0.0011	0.65 (0.37–1.14)	0.1299
• Depression						
-No (Ref)						
-Yes	0.60 (0.37–0.97)	0.0385	1.81 (1.07–3.04)	0.0261	0.96 (0.58–1.58)	0.8714
• Diabetes						
-No (Ref)						
-Yes	0.50 (0.31–0.81)	0.0051	1.32 (0.74–2.35)	0.3540	2.20 (1.15–4.22)	0.0176
• Hypertension						
-No (Ref)						
-Yes	0.83 (0.59–1.16)	0.2775	0.89 (0.61–1.30)	0.5533	1.42 (0.91–2.22)	0.1179
• Other co-morbidities						
-No (Ref)						
-Yes	1.09 (0.75–1.59)	0.6578	1.15 (0.77–1.72)	0.5079	0.66 (0.36–1.21)	0.1800

### Additional exploratory analysis

Depression continues to be a common mental health condition that could be misdiagnosed as a different psychiatric disorder leading to inappropriate treatment and potentially negative patient outcomes [6, 11, 36, 37]. We explored whether Black patients diagnosed with SSDs had higher odds than their White counterparts of being considered “depressed” (using the ‘depression’ question in the NAMCS). To do so, we conducted a bivariate analysis of race/ethnicity vs. depression. We found that 25.0% of White patients had screened as depressed, 27.8% of Black patients, 26.0% of Hispanic patients, and 24.1% for Asian/Others, which did not reach statistical significance. Post hoc tests (using the Bonferroni correction method) indicated no statistically significant difference among Blacks, Whites, and other race/ethnic groups, either. The results suggested no statistically significant overdiagnosis of SSDs in depressed Black care recipients (Data analysis not shown).

## Discussion

Analyzing data from the National Ambulatory Medical Care Survey from 2010 to 2015, we found that Black individuals were disproportionally represented in SSDs diagnoses (proportion 21.0% and rate 2.08%). Regarding specific SSDs, Black patients were also disproportionally represented with the schizophrenia, schizoaffective, and unspecified psychosis disorder (24.0%, 17.0%, and 26.0%, respectively). After controlling for the influence of confounding variables, our results revealed that compared to diagnoses received by White patients, Black patients had nearly twice the odds of receiving a schizophrenia diagnosis but were less likely to receive schizoaffective disorder diagnosis.

Considering that the non-Hispanic Black group alone represents 12.0% of the U.S. population, our findings support the US and European empirical reports of disproportionately high rates of SSDs in our sample [6, 9, 12, 13]. However, our data do not consider ethnoracial population differences in incidence rates of specific SSDs, so caution is warranted in interpreting whether these findings are due to etiologic or systemic issues as reported by prior investigators [6, 17, 25, 27, 38–40]. These investigators argue that individual upbringing, environment, acute and chronic discrimination, as well as structural racism, contributes to increased risk of trauma exposure and trauma severity, contributing to the development of SSDs.

Our reported estimates could suggest two possibilities. Either Black patients with SSDs more frequently use office-based outpatient treatment. Or that Black patients seeking mental health outpatient care receive a diagnosis of SSDs more often than White patients because of some other reason than “having” an SSD, such as misdiagnosis of bipolar and other mood disorders. Since NAMCA relies exclusively on clinicians’ own diagnoses and it is not clear what method the clinicians used to classify the ethnoracial identity of the participating patients, it is difficult to rely on either of these options fully. Trend analysis could help identify utilization over time.

Nevertheless, these interpretations should be given prominent attention since they could have disruptive implications such as contact with criminal justice [41], hospitalization [42], or suicide [43]. Overutilizing outpatient mental health care for SSDs could lead to unnecessary treatment or invasive procedures, impacting patient mental health outcomes [21]. Also, clinicians’ misdiagnoses or underdiagnoses of SSDs, could result in delayed treatment [44], inappropriate treatment [45], patient mistrust [22, 46, 47], missed diagnosis of comorbid mental health conditions such as depression [36, 37] or substance abuse [48, 49].

To address these implications, several strategies have been offered. These include promoting integrated mental health care that addresses systemic factors [50–52], disentangling institutional policies and practices that support structural racism [24, 53], supporting community initiatives that promote wellness [54], advocacy for changes to laws and policies that perpetuate racism and discrimination [55, 56], addressing adverse family experiences field [46, 49, 57, 58], and debiasing mental illness among mental health professionals [24, 59–64]. Others have suggested strategies to promote wellness and wellbeing by training a competent mental health workforce to practice and advocate for mainstreaming mental health promotion policies [54, 65]. In one focus group study, Blacks preferred using the term ‘emotional wellness’ instead of mental illness during their discussion. They addressed the importance of social support, availability, and accessibility to recreational facilities to promote mental health [46].

Additional noteworthy finding in our study is the significant proportion of Black patients with schizophrenia that were referred to other clinicians (21.2%) and that Black patients diagnosed with schizophrenia had a lower proportion of antipsychotic prescriptions (61.0%). These findings could suggest two scenarios while noting the limitations of the NAMCS data. One is that the participating clinicians chose to refer Black patients to other providers without at the same time initiating antipsychotic medication. Or that the clinicians are more uncertain of the SSDs diagnosis in the Black groups and unwilling to expose them to the medications without greater diagnostic certainty. Each option can potentially change the nature of the disparity and lines of investigation. In the first case scenario, it could delay the treatment [44], leading to contact with the criminal justice [41], hospitalization [42], and suicide [43]. In the second scenario, disparities in accessing psychotropic medications preclude this population from achieving optimum mental health, an issue that needs to be addressed [66]. Existing evidence shows that Black and Hispanic mental health service users are consistently and significantly less likely to receive clozapine than their White counterparts [67–69], even when favorable outcomes are reported [70–72], and also observed in minority patients [68].

### Limitations

While the main strength of the paper is the choice of topic, which is timely and important, given the growing attention to structural racism impacting access to mental health care and psychiatric diagnostic practice, our study has several limitations. Our data source, the National Ambulatory Medical Care Survey (NAMCS), was limited to 2010–2015. NAMCS is a complex dataset, which is an inherent limitation of most clinical epidemiology/service-use databases. NAMCS is office-based physician visits data that does not capture visits to hospital-affiliated clinics and emergency departments, which includes about 8.5% of all outpatient visits [73], indicating population bias. Also, NAMCS data needs to be clarified on the methods clinicians used to classify the ethnoracial identity of the patients. For example, it is not clear what proportion of the “Black” or “White” patients self-identified as “Hispanics.” This indeterminacy limits our certainty in associating differences in diagnoses with ethnoracial disparities. Also, the broad use of racial/ethnic groups did not allow us to capture factors such as country of origin or immigration status, which could impact treatment receiving [11]. Additionally, we cannot determine if the distribution of SSDs is due to a higher likelihood of overutilization of outpatient care or the psychiatric diagnostic practice of clinicians. The true reason is unknowable based on NAMCA data. Having a second source of diagnostic information could help clarify this issue.

Moreover, our study findings are limited to estimating proportions based on the number of visits made within a group of patients diagnosed with SSDs rather than considering population differences in the incidence of rates of disorders. Also, the sample size for the unspecified psychotic disorder category was smaller than the other diagnostic categories, which could account for the finding of fewer significant demographic differences in this sample. Furthermore, as the unit of sampling is a patient visit, disease prevalence cannot be directly determined [74]. This also limits our ethnonational interpretation of diagnostic differences. It should also be noted that NAMCS does not capture schizophrenia care data from other care settings, such as community mental health centers, other public facilities, and non-physician care.

Furthermore, as the NAMCS takes only a cross-section of visits to physicians’ offices, patients cannot be followed longitudinally to determine how their health changes over time. There is no direct information in the NAMCS on the outcomes of treatment. No disease severity measures are recorded. Moreover, non-adherent patients or patients with severe psychosis symptoms could be over-represented in the data since the survey counts visits, not individuals. It has been reported that Black patients are less likely to adhere to antipsychotic medications [75], which may lead to more ambulatory visits and crisis care, potentially introducing bias to the results [11].

## Conclusions

Our results show that SSDs, more specifically schizophrenia, continue to burden the mental health of Black individuals. Validation of our findings requires rigorous research at the population level that reveals the epidemiological difference of SSDs diagnoses in different race/ethnicity groups. Also, advancing our understanding of the nature of disparity in SSDs diagnoses among the Black population requires disentangling etiologic and systemic factors in play. This could include psychological stress, the pathway to care, services use, provider diagnostic practice, and experiencing discrimination and institutional and structural racism.

## Data Availability

The dataset analyzed (i.e., National Center for Health Statistics, Ambulatory Health Care Data) during the current study is publicly available and downloadable from https://www.cdc.gov/nchs/ahcd/datasets_documentation_related.htm, and also available from the corresponding author on reasonable request.

## References

[1] Kessler RC, Birnbaum H, Demler O, Falloon IR, Gagnon E, Guyer M (2005). The prevalence and correlates of nonaffective psychosis in the National Comorbidity Survey replication (NCS-R). Biol Psychiatry.

[2] Huang A, Amos TB, Joshi K, Wang L, Nash A (2018). Understanding healthcare burden and treatment patterns among young adults with schizophrenia. J Med Econ.

[3] Census.gov. 2017 [Available from: https://www.census.gov/quickfacts/fact/table/US/PST045218.

[4] Gunderson JG, Mosher LR (1975). The cost of schizophrenia. Am J Psychiatry.

[5] Cloutier M, Aigbogun MS, Guerin A, Nitulescu R, Ramanakumar AV, Kamat SA (2016). The Economic Burden of Schizophrenia in the United States in 2013. J Clin Psychiatry.

[6] Bourque F, van der Ven E, Malla A (2011). A meta-analysis of the risk for psychotic disorders among first- and second-generation immigrants. Psychol Med.

[7] Dealberto MJ (2010). Ethnic origin and increased risk for schizophrenia in immigrants to countries of recent and longstanding immigration. Acta Psychiatr Scand.

[8] Kirkbride JB, Errazuriz A, Croudace TJ, Morgan C, Jackson D, Boydell J (2012). Incidence of schizophrenia and other psychoses in England, 1950–2009: a systematic review and meta-analyses. PLoS ONE.

[9] Selten J-P, van der Ven E, Termorshuizen F (2020). Migration and psychosis: a meta-analysis of incidence studies. Psychol Med.

[10] Lazaridou FB, Schubert SJ, Ringeisen T, Kaminski J, Heinz A, Kluge U. Racism and psychosis: an umbrella review and qualitative analysis of the mental health consequences of racism. Eur Arch Psychiatry Clin Neurosci. 2022:1–14. 10.1007/s00406-022-01468-8 PMC9400567 36001139

[11] Manseau M, Case BG (2014). Racial-ethnic disparities in outpatient mental health visits to U.S. physicians, 1993–2008. Psychiatr Serv.

[12] Cantor-Graae E, Selten JP (2005). Schizophrenia and migration: a meta-analysis and review. Am J Psychiatry.

[13] McGrath J, Saha S, Welham J, El Saadi O, MacCauley C, Chant D (2004). A systematic review of the incidence of schizophrenia: the distribution of rates and the influence of sex, urbanicity, migrant status and methodology. BMC Med.

[14] Beards S, Gayer-Anderson C, Borges S, Dewey ME, Fisher HL, Morgan C (2013). Life events and psychosis: a review and meta-analysis. Schizophr Bull.

[15] Varese F, Smeets F, Drukker M, Lieverse R, Lataster T, Viechtbauer W (2012). Childhood adversities increase the risk of psychosis: a meta-analysis of patient-control, prospective- and cross-sectional cohort studies. Schizophr Bull.

[16] March D, Hatch SL, Morgan C, Kirkbride JB, Bresnahan M, Fearon P (2008). Psychosis and place. Epidemiol Rev.

[17] Pearce J, Rafiq S, Simpson J, Varese F (2019). Perceived discrimination and psychosis: a systematic review of the literature. Soc Psychiatry Psychiatr Epidemiol.

[18] Strakowski SM, Flaum M, Amador X, Bracha HS, Pandurangi AK, Robinson D (1996). Racial differences in the diagnosis of psychosis. Schizophr Res.

[19] Arnold LM, Keck PE, Collins J, Wilson R, Fleck DE, Corey KB (2004). Ethnicity and first-rank symptoms in patients with psychosis. Schizophr Res.

[20] Barrio C, Yamada A-M, Atuel H, Hough RL, Yee S, Berthot B (2003). A tri-ethnic examination of symptom expression on the positive and negative syndrome scale in schizophrenia spectrum disorders. Schizophr Res.

[21] Snowden LR (2003). Bias in mental health assessment and intervention: theory and evidence. Am J Public Health.

[22] Eack SM, Bahorik AL, Newhill CE, Neighbors HW, Davis LE (2012). Interviewer-perceived honesty as a mediator of racial disparities in the diagnosis of schizophrenia. Psychiatr Serv.

[23] Gara MA, Vega WA, Arndt S, Escamilla M, Fleck DE, Lawson WB (2012). Influence of patient race and ethnicity on clinical assessment in patients with affective disorders. Arch Gen Psychiatry.

[24] Bailey ZD, Feldman JM, Bassett MT (2021). How structural Racism Works - Racist Policies as a Root cause of U.S. racial health inequities. N Engl J Med.

[25] Anglin DM, Greenspoon M, Lighty Q, Ellman LM (2016). Race-based rejection sensitivity partially accounts for the relationship between racial discrimination and distressing attenuated positive psychotic symptoms. Early Interv Psychiatry.

[26] Williams DR (2018). Stress and the Mental Health of populations of Color: advancing our understanding of race-related stressors. J Health Soc Behav.

[27] Anglin DM, Ereshefsky S, Klaunig MJ, Bridgwater MA, Niendam TA, Ellman LM (2021). From womb to Neighborhood: a racial analysis of Social Determinants of psychosis in the United States. Am J Psychiatry.

[28] Bao Y, Fisher J, Studnicki J (2008). Racial differences in behavioral inpatient diagnosis: examining the mechanisms using the 2004 Florida Inpatient Discharge Data. J Behav Health Serv Res.

[29] Statistics NCfH. Center for Disease Control and Prevention. Ambulatory health care data. 2017 [Available from: https://www.cdc.gov/nchs/ahcd/index.htm.

[30] Grant RK, Brindle WM, Donnelly MC, McConville PM, Stroud TG, Bandieri L (2022). WJG World J Gastroenterol.

[31] Ventura AMB, Hayes RD, Fonseca de Freitas D (2022). Ethnic disparities in clozapine prescription for service-users with schizophrenia-spectrum disorders: a systematic review. Psychol Med.

[32] Nishanth KN, Chadda RK, Sood M, Biswas A, Lakshmy R (2017). Physical comorbidity in schizophrenia & its correlates. Indian J Med Res.

[33] Carney CP, Jones L, Woolson RF (2006). Medical comorbidity in women and men with schizophrenia. J Gen Intern Med.

[34] Jeste DV, Gladsjo JA, Lindamer LA, Lacro JP (1996). Medical comorbidity in schizophrenia. Schizophr Bull.

[35] Bhattacharjee S, Vadiei N, Goldstone L, Alrabiah Z, Sherman SJ (2018). Patterns and predictors of Depression Treatment among older adults with Parkinson’s Disease and Depression in Ambulatory Care Settings in the United States. Parkinsons Dis.

[36] Bailey RK, Blackmon HL, Stevens FL (2009). Major depressive disorder in the african american population: meeting the challenges of stigma, misdiagnosis, and treatment disparities. J Natl Med Assoc.

[37] Pederson AB (2023). Management of depression in black people: effects of cultural issues. Psychiatric Annals.

[38] van Os J, Kenis G, Rutten BP (2010). The environment and schizophrenia. Nature.

[39] Williams DR, González HM, Neighbors H, Nesse R, Abelson JM, Sweetman J (2007). Prevalence and distribution of major depressive disorder in African Americans, Caribbean blacks, and non-hispanic whites: results from the National Survey of American Life. Arch Gen Psychiatry.

[40] Misra S, Etkins OS, Yang LH, Williams DR (2022). Structural racism and inequities in incidence, course of illness, and treatment of psychotic Disorders among Black Americans. Am J Public Health.

[41] Seltzer T (2005). Mental health courts: a misguided attempt to address the criminal justice system’s unfair treatment of people with mental illnesses. Psychol Public Policy Law.

[42] Oluwoye O, Davis B, Kuhney FS, Anglin DM (2021). Systematic review of pathways to care in the U.S. for Black individuals with early psychosis. NPJ Schizophr.

[43] Joe S, Baser RE, Breeden G, Neighbors HW, Jackson JS (2006). Prevalence of and risk factors for lifetime suicide attempts among blacks in the United States. JAMA.

[44] Thornicroft G, Rose D, Kassam A (2007). Discrimination in health care against people with mental illness. Int Rev psychiatry.

[45] Akinhanmi MO, Biernacka JM, Strakowski SM, McElroy SL, Balls Berry JE, Merikangas KR (2018). Racial disparities in bipolar disorder treatment and research: a call to action. Bipolar Disord.

[46] Haynes TF, Cheney AM, Sullivan JG, Bryant K, Curran GM, Olson M (2017). Addressing Mental Health needs: perspectives of African Americans living in the Rural South. Psychiatr Serv.

[47] Suite DH, La Bril R, Primm A, Harrison-Ross P (2007). Beyond misdiagnosis, misunderstanding and mistrust: relevance of the historical perspective in the medical and mental health treatment of people of color. J Natl Med Assoc.

[48] McKetin R, Dawe S, Burns RA, Hides L, Kavanagh DJ, Teesson M (2016). The profile of psychiatric symptoms exacerbated by methamphetamine use. Drug Alcohol Depend.

[49] Bell CC, Jackson WM, Bell BH (2015). Misdiagnosis of African-Americans with Psychiatric Issues – Part II. J Natl Med Assoc.

[50] Sacks S, Chaple M, Sirikantraporn J, Sacks JY, Knickman J, Martinez J (2013). Improving the capability to provide integrated mental health and substance abuse services in a state system of outpatient care. J Subst Abuse Treat.

[51] Snowden LR, Thomas K (2000). Medicaid and African American outpatient mental health treatment. Ment Health Serv Res.

[52] Satcher D, Rachel SA (2017). Promoting mental health equity: the role of integrated care. J Clin Psychol Med Settings.

[53] Griffith DM, Mason M, Yonas M, Eng E, Jeffries V, Plihcik S (2007). Dismantling institutional racism: theory and action. Am J Community Psychol.

[54] Battel-Kirk B, Barry MM, Taub A, Lysoby L (2009). A review of the international literature on health promotion competencies: identifying frameworks and core competencies. Glob Health Promot.

[55] Estroff SE, Penn DL, Toporek JR (2004). From stigma to discrimination: an analysis of community efforts to reduce the negative consequences of having a psychiatric disorder and label. Schizophr Bull.

[56] Clery P, Embliss L, Cussans A, Cooke E, Shukla K, Li C (2022). Protesting for public health: a case for medical activism during the climate crisis. Int Rev Psychiatry.

[57] Ghoreishi A, Kabootvand S, Zangani E, Bazargan-Hejazi S, Ahmadi A, Khazaie H (2015). Prevalence and attributes of criminality in patients with schizophrenia. J Inj Violence Res.

[58] Memon A, Taylor K, Mohebati LM, Sundin J, Cooper M, Scanlon T (2016). Perceived barriers to accessing mental health services among black and minority ethnic (BME) communities: a qualitative study in Southeast England. BMJ Open.

[59] Kales HC, Neighbors HW, Valenstein M, Blow FC, McCarthy JF, Ignacio RV (2005). Effect of race and sex on primary care physicians’ diagnosis and treatment of late-life depression. J Am Geriatr Soc.

[60] Hairston DR, Gibbs TA, Wong SS, Jordan A (2019). Clinician bias in diagnosis and treatment.

[61] Schwartz RC, Feisthamel KP (2009). Disproportionate diagnosis of mental disorders among african american versus european american clients: implications for counseling theory, research, and practice. J Couns Dev.

[62] Schwartz RC, Blankenship DM (2014). Racial disparities in psychotic disorder diagnosis: a review of empirical literature. World J Psychiatry.

[63] Neighbors HW, Trierweiler SJ, Ford BC, Muroff JR (2003). Racial differences in DSM diagnosis using a semi-structured instrument: the importance of clinical judgment in the diagnosis of African Americans. J Health Soc Behav.

[64] Schwartz EK, Docherty NM, Najolia GM, Cohen AS (2019). Exploring the racial diagnostic bias of schizophrenia using behavioral and clinical-based measures. J Abnorm Psychol.

[65] Barry MM (2009). Addressing the determinants of positive Mental Health: concepts, evidence and practice. Int J Mental Health Promotion.

[66] World Health Organisation. International Statistical Classification of Diseases and Related Health Problems (ICD) 2019 [Available from: standards/classifications/classification-of-diseases.

[67] Das-Munshi J, Bhugra D, Crawford MJ (2018). Ethnic minority inequalities in access to treatments for schizophrenia and schizoaffective disorders: findings from a nationally representative cross-sectional study. BMC Med.

[68] Kelly DL, Dixon LB, Kreyenbuhl JA, Medoff D, Lehman AF, Love RC (2006). Clozapine utilization and outcomes by race in a public mental health system: 1994–2000. J Clin Psychiatry.

[69] Beck K, McCutcheon R, Stephenson L, Schilderman M, Patel N, Ramsay R (2019). Prevalence of treatment-resistant psychoses in the community: a naturalistic study. J Psychopharmacol.

[70] Baig AI, Bazargan-Hejazi S, Ebrahim G, Rodriguez-Lara J (2021). Clozapine prescribing barriers in the management of treatment-resistant schizophrenia: a systematic review. Medicine.

[71] Farooq S, Choudry A, Cohen D, Naeem F, Ayub M (2019). Barriers to using clozapine in treatment-resistant schizophrenia: systematic review. BJPsych Bull.

[72] Kesserwani J, Kadra G, Downs J, Shetty H, MacCabe JH, Taylor D (2019). Risk of readmission in patients with schizophrenia and schizoaffective disorder newly prescribed clozapine. J Psychopharmacol.

[73] Rhee TG, Mohamed S, Rosenheck RA. Antipsychotic prescriptions among adults with Major Depressive Disorder in Office-Based outpatient settings: National Trends from 2006 to 2015. J Clin Psychiatry. 2018;79(2). 10.4088/JCP.17m11970 PMC5932223 29469245

[74] Ahn CS, Allen M-M, Davis SA, Huang KE, Fleischer AB, Feldman SR (2014). The National Ambulatory Medical Care Survey: a resource for understanding the outpatient dermatology treatment. J Dermatological Treat.

[75] Nikolitch K, Ryder A, Jarvis GE. Adherence to Follow-Up in First-Episode Psychosis: ethnicity factors and case manager perceptions. Prim Care Companion CNS Disord. 2018;20(3). 10.4088/PCC.17m02156 29949246

